# Sedentary Behavior and Physical Activity of Community-Dwelling Korean Breast Cancer Survivors: A Nationwide Study

**DOI:** 10.3390/healthcare11131974

**Published:** 2023-07-07

**Authors:** Jung Soo Lee, Mina Park, Yeo Hyung Kim

**Affiliations:** Department of Rehabilitation Medicine, College of Medicine, The Catholic University of Korea, Seoul 06591, Republic of Korea; drlee71@catholic.ac.kr (J.S.L.); mmaabb@catholic.ac.kr (M.P.)

**Keywords:** sedentary behavior, exercise, physical fitness, neoplasms, breast neoplasms

## Abstract

This study aimed to investigate the contemporary characteristics of sedentary behavior and physical activity levels in breast cancer survivors. The cross-sectional data of 10,073 community-dwelling Korean women aged ≥50 years in the Korea National Health and Nutrition Examination Survey were analyzed. The differences in sedentary behavior, walking activity, and moderate-to-vigorous physical activity (MVPA) levels between breast cancer survivors, other cancer survivors, and women with no history of cancer were compared by complex-sample general linear models. Breast cancer survivors spent significantly less mean time in sedentary behavior than other cancer survivors and women with no history of cancer; however, among them, 48.34% spent a long sedentary time of ≥420 min/day. Breast cancer survivors had a significantly higher level of walking activity and similar total MVPA levels compared to women with no history of cancer. When comparing domain-specific MVPA levels, breast cancer survivors showed significantly lower work-related MVPA levels than women with no history of cancer. In recent years, community-dwelling Korean breast cancer survivors were less sedentary, walked more, and had equivalent MVPA levels compared with women with no history of cancer. Considering the growing emphasis on healthy lifestyles, our results may reflect more contemporary behavior trends of breast cancer survivors.

## 1. Introduction

Physical activity is defined as any bodily movement produced by skeletal muscles that requires energy expenditure, either for leisure, transportation, or work [1]. Physical activity has various beneficial effects on health outcomes, such as reducing all-cause mortality, contributing to social relationships and mental health, and preventing many chronic illnesses, including cancer [2]. The lack of participation in physical activity leads to sedentary behavior, which has a negative impact on health outcomes independently of physical activity [3]. Furthermore, there has been growing evidence that spending less time in sedentary behavior is beneficial for health, even among those with modest levels of physical activity. Despite the numerous documented health benefits, aging and chronic health conditions lead to a decrease in physical activity and an increase in sedentary behavior among middle-aged and older people in the community.

The benefits of physical activity in patients with cancer and in the general population have been well established by many previous studies [4]. Regular physical activity reduces the risk of colon and breast cancer [2]. Nevertheless, a lack of physical activity and an increase in sedentary behavior among cancer survivors are often mentioned as considerable health problems. Although patients with cancer have increased awareness of light physical activity as well as moderate-to-vigorous physical activity, various potential barriers may contribute to their low levels of physical activity, such as cancer therapy-related side effects, fear of motion, and the lack of appropriately tailored facilities, which would be different across various countries [5,6].

Many breast cancer survivors suffer from impairments of the upper extremities, depression, or cognitive decline after treatment, which leads to limitations in activities of daily living and participation in work, sports, and leisure activities [7,8]. These impairments and adverse effects can act as potential barriers to physical activity in breast cancer survivors. Earlier studies have reported that pre-operative total physical activity levels and occupational, sport, and household activity levels are not recovered after breast cancer surgery [9]. A cohort study showed that physical activity levels during and after treatment in patients with breast cancer were lower than those in women of similar age in the general population [10]. However, to the best of our knowledge, research regarding the characteristics of sedentary behavior and physical activity levels in Asian breast cancer survivors has rarely been reported.

Considering the increasing incidence and survival rate of patients with breast cancer, the role of physical activity among breast cancer survivors in leading a healthy life has been increasingly emphasized [11]. Nevertheless, research on the contemporary status of physical activity and sedentary behavior in community-dwelling breast cancer survivors, especially in Asian populations, is lacking. Therefore, the present study aimed to investigate the characteristics of sedentary behavior and physical activity levels in community-dwelling Korean breast cancer survivors aged 50 years or older, compared with survivors of other types of cancer and those without a history of cancer. Given the recent increase in public interest in a healthy lifestyle and the consistent emphasis on regular exercise at cancer clinics [12,13,14], we hypothesized that breast cancer survivors in recent years may be less sedentary or more physically active than previously estimated.

## 2. Materials and Methods

### 2.1. Study Design and Participants

This cross-sectional study utilized the data from the Korea National Health and Nutrition Examination Survey (KNHANES) VI, VII, and VIII [15]. The Korea Disease Control and Prevention Agency annually collects comprehensive health-related data for KNHANES through household interviews and quality-controlled physical examinations. The KNHANES collects information on health behavior, the prevalence of chronic diseases, and the status of food and nutritional intake among the community-dwelling Korean population. The KNHANES applies a multi-stage, stratified, cluster sampling strategy to gather a nationally representative sample of the Korean population. The detailed data resource profile has been published previously [16]. The participation rates of the KNHANES cycles were reported as follows: 78.3% for cycle VI, 76.6% for cycle VII, and 74.0% for cycle VIII. Anonymized KNHANES data and user guidelines are publicly accessible on a website in Korean (https://knhanes.kdca.go.kr (accessed on 29 November 2022)). All participants of the KNHANES provided informed consent before participation, and the requirement for ethical approval was exempted by the Institutional Review Board of our hospital because the current study uses the publicly available data of the KNHANES.

A total of 47,309 participants (25,743 females) were recruited for the KNHANES between 2014 and 2019. Among the total participants, 20,546 participants (11,658 females) were aged 50 years or older (43.43%). After excluding people with a missing history of cancer (*n* = 1102) and with double primary cancer in the breast and other organs (*n* = 12), 10,073 female participants were finally included in the current study. Women who had ever been diagnosed with cancer by a doctor were defined as cancer survivors [17]. In the present study, three groups of women were compared: survivors of breast cancer (*n* = 193), survivors of cancers other than breast cancer (*n* = 644), and those without a history of cancer (9236) [18]. Because we excluded the participants with double primary cancers in the breast and other organs, we were able to categorize study participants exclusively into one of these three groups.

### 2.2. Physical Activity Assessment

Sedentary behavior and moderate-to-vigorous physical activity were evaluated using a validated Korean version of the World Health Organization (WHO) Global Physical Activity Questionnaire (GPAQ) [19]. The GPAQ gathers physical activity data in three domains (work, travel, and recreational activities), as well as sedentary behavior. The time spent in moderate-to-vigorous physical activity is obtained from the codes for the frequency (days/week) and duration (min/day) spent engaged in physical activity in a typical week. The intensity of physical activities is expressed in metabolic equivalents of tasks (METs) (MET-min/week), calculated by multiplying time spent in physical activity (min/week) by 4.0 METs for moderate activities or 8.0 METs for vigorous activities. The total moderate-to-vigorous physical activity level was categorized into three levels (high, moderate, or low) according to the GPAQ analysis guide [19]. Long sedentary time was defined as spending ≥420 min/day sedentary [20], and participants with long sedentary time were classified as sedentary individuals.

Because the information on walking and resistance exercise is not obtainable via the GPAQ, walking activity and resistance exercise were separately surveyed [21]. Walking activity was assessed by questions 5 and 6 of the Korean version of the International Physical Activity Questionnaire Short Form [22]. Time spent walking for more than 10 continuous minutes in the last 7 days across all contexts (work, household walking, travel, recreation, exercise, or leisure) was recorded. Because the above questions include light walking, which does not cause an increase in breathing or heart rate, a value of 3.3 METs for walking is recommended. The intensity of walking was categorized into tertiles: highest (>693 MET-min/week), middle (199–693 MET-min/week), or lowest (≤198 MET-min/week). The participants were also asked how many days during the last week they did resistance exercises, such as push-ups, dumbbells, or barbells. Sufficient resistance exercise was defined as ≥2 days/week, according to the WHO guideline [2].

### 2.3. Variables

Height was measured with the minimum requirement that the heels and buttocks touched the vertical board while standing. Weight was measured in a state of exhaling breath, wearing light clothing only. Body mass index was calculated using measured weight (kg) divided by height squared (m^2^). Information on education level (>9 years or ≤9 years) and residence area (urban or rural) was obtained. Those who worked for more than 1 h in the past week with the purpose of earning income or worked as unpaid family workers for more than 18 h were considered to have an occupation (employed state). Individuals who had never consumed alcohol or had not consumed alcohol at all in the last year were classified as non-drinkers.

Individuals with a systolic blood pressure ≥140 mmHg, a diastolic blood pressure ≥90 mmHg, or those who took antihypertensive medications were considered hypertensive participants. Women with a fasting blood glucose ≥126 mg/dL, hemoglobin A1c ≥ 6.5%, diagnosed as having diabetes by a doctor, or using hypoglycemic agents or insulin injections were considered to have diabetes. Hypercholesterolemia was classified as participants with total cholesterol ≥240 mg/dL or who were taking cholesterol-lowering drugs. Women with hemoglobin ≤12 g/dL were defined as having anemia. Participants who had been diagnosed with cardiovascular disease or depression by a doctor were defined as having cardiovascular disease or depression, respectively.

### 2.4. Statistical Analysis

The collected data from the KNHANES comprised the sample survey with a complex sampling design, which means that sample data have an unequal probability of response and dropout rates. Therefore, it is strongly recommended that sampling weights should be calculated and used to correct the bias, such as from missing data, unequal sampling rates, and non-response errors. During the process of analyzing the data from the KNHANES, by applying sampling weights, the inclusion errors due to differences in the number of households and populations between the sampling design stage and the survey stage, unequal sampling rates, and non-response errors of non-participants in the survey can be adjusted. As a result, the analysis results can be interpreted as representative results of the entire target population, which in this case is the Korean population, with high accuracy. Therefore, in all statistical analyses of the present study, we applied the weighted values calculated from sampling weights, non-response-adjusted weights using estimated response probability, and calibration weights considering the clustering and stratification of the sample survey data.

The characteristics of participants by cancer status (breast cancer survivors, other cancer survivors, and no history of cancer) were compared using the complex-sample Student’s *t*-test for continuous variables and the complex-sample Chi-square test for categorical variables. The prevalence of sedentary behavior, walking activity, moderate-to-vigorous physical activity, and resistance exercise levels between the three groups by cancer status were analyzed using the complex-sample Chi-square test. Mean daily time spent in sedentary behavior and mean weekly METs spent in walking and moderate-to-vigorous physical activity were calculated using complex-sample general linear models, adjusting for multiple potential covariables. Data were presented as weighted mean ± standard error or weighted percentage (standard error), as appropriate. All statistics were calculated using the Statistical Package for the Social Sciences, version 22 (IBM Corp., Armonk, NY, USA). *p*-values < 0.05 were considered statistically significant.

## 3. Results

Among the 10,073 women aged 50 years or older, the prevalence of breast cancer survivors, other cancer survivors, and individuals with no history of cancer was 1.88%, 6.65%, and 91.47%, respectively. Characteristics of participants are compared by cancer status in Table 1. Participants with no history of cancer were more likely to have a higher body mass index than breast and other cancer survivors. The proportion of educational status, occupational status, alcohol consumption, and anemia differed significantly between breast cancer survivors and other cancer survivors. The mean age and the proportion of residence area, hypertension, diabetes, hypercholesterolemia, cardiovascular disease, and depression were not significantly different across the breast cancer survivors, other cancer survivors, and individuals with no history of cancer.

**Figure 1:**
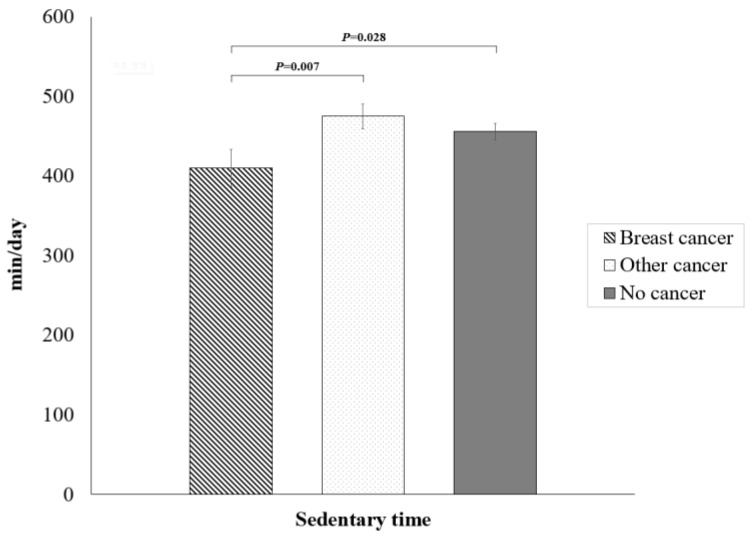
Mean daily time of sedentary behavior in community-dwelling women aged 50 years or older by cancer status. Values are adjusted for age, body mass index, education, occupation, residence, alcohol consumption, hypertension, diabetes, hypercholesterolemia, anemia, cardiovascular disease, and depression.

**Figure 2:**
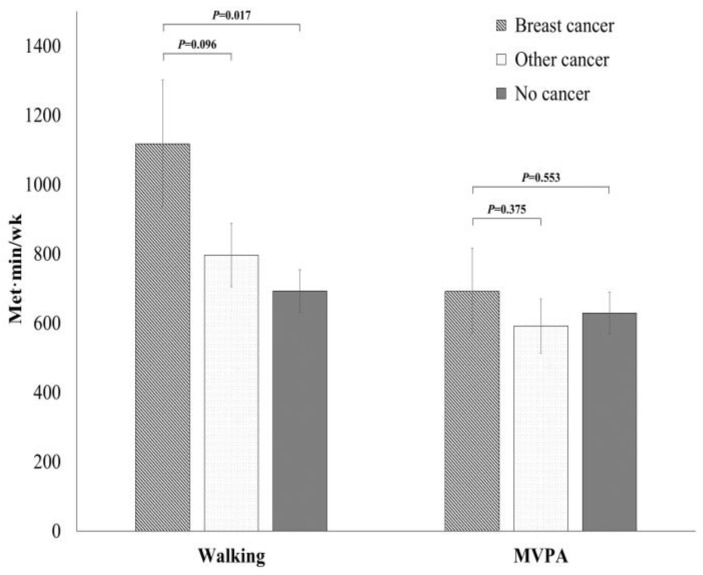
Mean weekly METs of walking/MVPA in community-dwelling women aged 50 years or older by cancer status. Values are adjusted for age, body mass index, education, occupation, residence, alcohol consumption, hypertension, diabetes, hypercholesterolemia, anemia, cardiovascular disease, and depression. MVPA, moderate-to-vigorous physical activity; MET, metabolic equivalent of task.

Table 2 presents the sedentary behavior and physical activity pattern by cancer status. The prevalence of sedentary individuals (48.34%) was significantly lower in breast cancer survivors than in other cancer survivors (54.92%). Furthermore, breast cancer survivors spent significantly less time in sedentary behavior (409.92 ± 23.59 min/day) than other cancer survivors (474.84 ± 15.76 min/day) and participants with no history of cancer (455.60 ± 10.21 min/day), after adjusting for multiple potential confounders (Figure 1). Further, as shown in Table 2, the prevalence of the highest walking tertile was significantly higher in breast cancer survivors (47.91%) than in other cancer survivors (36.69%) and individuals with no history of cancer (33.07%). Figure 2 also shows that breast cancer survivors walked significantly more (1117.77 ± 184.65 MET-min/week) than participants with no history of cancer (692.80 ± 61.38 MET-min/week).

Meanwhile, the levels of total moderate-to-vigorous physical activity and resistance exercise were not significantly different among breast cancer survivors, other cancer survivors, and participants with no history of cancer (Table 2). The mean weekly METs spent in moderate-to-vigorous physical activity were also similar between the three groups as shown in Figure 2. However, when compared according to the domain-specific moderate-to-vigorous physical activity, breast cancer survivors had lower work-related moderate-to-vigorous physical activity levels than participants with no history of cancer (Table 3). There was no significant difference in travel and recreational moderate-to-vigorous physical activity among breast cancer survivors, other cancer survivors, and individuals with no history of cancer.

## 4. Discussion

The current study presents the contemporary sedentary behavior and physical activity patterns in community-dwelling Korean breast cancer survivors aged 50 years or older. Breast cancer survivors spent less sedentary time than other cancer survivors and participants with no history of cancer. Furthermore, breast cancer survivors walked significantly more than females without a history of cancer. However, total moderate-to-vigorous physical activity levels were similar between survivors of breast cancer, survivors of cancers other than breast cancer, and those without a history of cancer. With regard to domain-specific moderate-to-vigorous physical activity, travel- and recreation-related moderate-to-vigorous physical activity were not significantly different across the three groups, but work-related moderate-to-vigorous physical activity was significantly lower in the survivors of breast and other cancers than in the participants without a history of cancer.

Regarding our findings that Korean breast cancer survivors spent less sedentary time than the non-cancer controls and other cancer survivors, the results of our study differed from earlier studies reporting that breast cancer survivors are more sedentary than women who did not have cancer [23,24,25]. However, contrary to the earlier studies, more recent studies have begun to report findings that breast cancer survivors spend equivalent or less sedentary time compared with controls with no history of cancer [26,27]. A recent cohort of breast cancer survivors in Canada reported similar levels of sedentary time compared to the general population of women in the United States [28]. Considering the continuous emphasis on non-sedentary lifestyles in cancer clinics and the increasing interest in physical activity and healthy lifestyles by the modern population [12], the results of our study may reflect more contemporary behavior trends of breast cancer survivors. Meanwhile, since most existing evidence on the sedentary behavior of breast cancer survivors stems from the results of studies in the United States or Australia, the results of the current research reflect that the lifestyle of Asians may differ.

While breast cancer survivors were less sedentary than females without a history of cancer and survivors of other cancers, approximately half of them still spent a relatively long time in sedentary behavior—over 7 h per day. Given that the prevalence of sedentary individuals is still high among breast cancer survivors, community-based tailored physical activity-promoting programs, incorporating moderate-to-vigorous physical activity as well as simple walking, are necessary. Recent studies suggest that sedentary behavior has a negative impact on health outcomes that are independent of the overall amount of physical activity, and that spending more time in physical activity cannot reduce the detrimental effect of prolonged sedentary behavior on health outcomes [29]. The WHO guidelines on physical activity and sedentary behavior recommend that sedentary behavior should be limited across all groups and that replacing sedentary time with any intensity of physical activity, including light-intensity physical activity, has significant health benefits [1]. Given the fact that reducing the amount of sedentary behavior itself is important for health outcomes in breast cancer survivors [30,31], the current study suggests that tailored promotion strategies to reduce prolonged sedentary behavior are needed in community-dwelling breast cancer survivors.

The results of our study indicated that community-dwelling breast cancer survivors walked significantly more than women without a history of cancer. This notable result is encouraging for public health, given that walking is the most easily accessible and a relatively light form of physical activity [25]. To our knowledge, previous studies comparing walking behaviors between breast cancer survivors and non-cancer controls are rare. The results of a recent systematic review that walking was the strongest physical activity modality preference among cancer survivors may support the results of our study [13]. Given that walking is usually regarded as light physical activity without a significant increase in breath or heart rate, the results of our study are contrary to those of previous studies that showed no difference in light physical activity between breast cancer survivors and non-cancer controls [24,26]. This discrepancy between our study and previous studies may be because the inclusion of faster speeds of walking with higher intensities, such as Nordic walking, varies across the studies [32].

Inconsistency persists regarding the moderate-to-vigorous physical activity levels in breast cancer survivors compared with controls [10,24,26]. The results of the current study showing that the moderate-to-vigorous physical activity level was similar between breast cancer survivors, non-cancer controls, and other cancer survivors are consistent with a cohort study in the Netherlands reporting that physical activity guideline adherence from 12 months post-treatment was similar between women with breast cancer and the Dutch female population without breast cancer [10]. Another study in the United States found that breast cancer survivors aged over 60 years spent only an estimated 2.6% of their waking time in moderate-to-vigorous physical activity, although they engaged in more moderate-to-vigorous physical activity than controls (2.6% vs. 1.8%) [23]. Because breast cancer survivors tend to spend remarkably less time in moderate-to-vigorous physical activity than light physical activity, studies may have reported inconsistent results for moderate-to-vigorous physical activity according to the study population.

The results of our study that breast cancer survivors were sedentary, walked more, and performed similar moderate-to-vigorous physical activity compared to controls may reflect the physical modality preference of breast cancer survivors. A pilot randomized controlled trial found that reductions in sedentary time were greater in participants randomized to the lower-intensity physical activity group than in those randomized to the higher-intensity physical activity group and control group [33]. A cross-sectional study suggested fatigue, pain, and discomfort as major barriers to being physically active among breast cancer survivors [34]. However, the barriers to moderate-to-vigorous physical activity and exercise preference may differ across cancer centers, countries, races, and cultures [14]. Since evidence of the benefits of reducing sedentary behavior in breast cancer survivors is accumulating, promoting an increase in light-intensity physical activities, such as simple walking, has emerged as a practical approach, especially in older breast cancer survivors [35].

As with the previous literature reporting reduced work engagement and work ability in breast cancer survivors [36], our study found significantly lower work-related moderate-to-vigorous physical activity in breast cancer survivors than in women without a history of cancer. Although the survival rate of people diagnosed with breast cancer increases with the advancement of early detection and treatment methods, there are various known barriers to returning to work for breast cancer survivors [37]. Lymphedema and upper limb pain and discomfort, which affect many breast cancer survivors, can adversely affect the usage of the upper limbs, deteriorating work function and endurance, including moderate-to-vigorous physical activities. Furthermore, the symptom clusters, including pain, fatigue, and psychological distress of long-term breast cancer survivors, might have a negative impact on returning to work and work endurance [38].

The strength of our study is that it presents contemporary real-world data from breast cancer survivors living in the Korean community, rather than hospitalized patients under treatment for breast cancer. Since research on the physical activity and sedentary behavior of Asian breast cancer survivors is lacking, our study provides valuable insight into the behavioral patterns of middle-aged and older Asian female breast cancer survivors. Therefore, the results of this study may suggest the future direction for interventions to reduce sedentary behavior and promote physical activity in breast cancer survivors living in the community. The limitations of our study stem from the cross-sectional design of the KNHANES, which cannot reveal the causal relationships between variables, such as the association between physical activity and breast cancer. Second, the self-reported surveys of physical activity and sedentary behavior could confer recall bias. In addition, the data obtained by self-reported questionnaires and objective physical activity measures may show different results. Third, because there are no detailed cancer-related data in the KNHANES, such as the stage and treatment status, their effects are unknown. Moreover, since the KNHANES does not include information on participants prior to cancer diagnosis, it was not possible to adjust for pre-cancer factors during the analyses. Finally, considering the differences in demographic and cancer-related factors across the various countries, the results of our study may not be generalizable to other populations.

## 5. Conclusions

Community-dwelling Korean breast cancer survivors aged 50 years or older were less sedentary and walked more than women without a history of cancer. However, moderate-to-vigorous physical activity levels were similar between breast cancer survivors, other cancer survivors, and women with no history of cancer. Community-dwelling breast cancer survivors may prefer walking as a mode of physical activity in order to be less sedentary. Therefore, future prospective studies and randomized controlled trials are needed to examine the factors associated with the types of physical activity chosen by breast cancer survivors to reduce sedentary behavior. By synthesizing the findings of our study and future studies, personalized programs can be developed to convert sedentary time into physical activity in community-dwelling breast cancer survivors. In addition, healthcare professionals should regularly recommend increasing physical activity, assuming that half of the breast cancer survivors spend a long time being sedentary.

## Figures and Tables

**Table 1:** Characteristics of community-dwelling females aged ≥ 50 years by cancer status.

	Breast Cancer Survivors	Other Cancer Survivors	No History of Cancer	*p* Value *
Unweighted number	193	644	9236	
Weighted number	163,624	578,053	7,950,902	
Age (years)	62.38 ± 0.79	63.60 ± 0.42	62.85 ± 0.13	0.199
Body mass index (kg/m^2^)	23.89 ± 0.32	23.68 ± 0.13	24.13 ± 0.04	0.004
Education				0.001
>9 years	57.21 (4.12)	42.96 (2.27)	41.75 (0.78)	
≤9 years	42.79 (4.12)	57.04 (2.27)	58.25 (0.78)	
Occupation				<0.001
Employed	37.28 (4.06)	36.07 (2.27)	46.01 (0.70)	
Unemployed	62.72 (4.06)	63.93 (2.27)	53.99 (0.70)	
Residence				0.389
Urban	84.60 (2.99)	81.76 (1.95)	80.69 (1.22)	
Rural	15.40 (2.99)	18.24 (1.95)	19.31 (1.22)	
Alcohol				<0.001
Drinker	55.94 (5.11)	63.64 (2.48)	71.14 (0.62)	
Non-drinker	44.06 (5.11)	36.36 (2.48)	28.86 (0.62)	
Comorbidities				
Hypertension	40.73 (3.93)	43.29 (2.24)	45.41 (0.64)	0.349
Diabetes	19.06 (4.10)	13.21 (1.79)	15.80 (0.54)	0.284
Hypercholesterolemia	33.56 (4.37)	40.16 (2.47)	36.81 (0.62)	0.297
Anemia	17.19 (3.78)	13.57 (1.73)	8.95 (0.36)	<0.001
Cardiovascular disease	3.72 (1.26)	6.57 (1.13)	6.25 (0.29)	0.377
Depression	8.42 (2.24)	7.76 (1.12)	7.85 (0.33)	0.963

Values are weighted mean ± standard error or weighted percentage (standard error), as appropriate. * *p* values using Student’s *t*-test or Chi-square test, as appropriate.

**Table 2:** Weighted prevalence of sedentary behavior and physical activity in study participants by cancer status.

	Breast Cancer Survivors	Other Cancer Survivors	No History of Cancer	*p* Value *
Sedentary behavior				0.044
Non-sedentary	51.66 (4.30)	45.08 (2.32)	50.94 (0.67)	
Sedentary	48.34 (4.30)	54.92 (2.32)	49.06 (0.67)	
Walking tertile				0.002
Highest	47.91 (4.21)	36.69 (2.10)	33.07 (0.60)	
Middle	28.28 (3.68)	31.31 (2.10)	33.39 (0.58)	
Lowest	23.81 (3.47)	31.99 (2.06)	33.54 (0.66)	
Total MVPA level				0.514
High	5.50 (1.77)	4.56 (0.88)	4.51 (0.26)	
Moderate	33.08 (4.05)	29.37 (2.10)	27.69 (0.57)	
Low	61.42 (4.18)	66.07 (2.15)	67.80 (0.61)	
Resistance exercise				0.287
Sufficient	16.79 (3.06)	13.69 (1.54)	12.68 (0.43)	
Insufficient	83.21 (3.06)	86.31 (1.54)	87.32 (0.43)	

Values are weighted percentage (standard error). * *p* values using Chi-square test. MVPA, moderate-to-vigorous physical activity.

**Table 3:** Mean weekly METs spent in three domains of MVPA in study participants by cancer status.

MVPA Domains (MET-Min/Week)	Breast Cancer Survivors (BC)	Other Cancer Survivors (OC)	No History of Cancer (NC)	*p* Value *	*p* Value ^†^		
					BC vs. OC	BC vs. NC	OC vs. NC
Work	82.51 ± 36.13	89.57 ± 39.77	150.79 ± 40.37	<0.001	0.641	<0.001	<0.001
Travel	493.51 ± 88.37	421.27 ± 58.89	386.21 ± 38.49	0.318	0.385	0.158	0.451
Recreational	116.55 ± 85.45	81.02 ± 32.74	91.81 ± 18.04	0.889	0.685	0.770	0.713

Values are adjusted for age, body mass index, education, occupation, residence, alcohol consumption, hypertension, diabetes, hypercholesterolemia, anemia, cardiovascular disease, and depression. * *p* values using general linear models. ^†^ *p* values using post hoc analyses. MVPA, moderate-to-vigorous physical activity; METs, metabolic equivalents of tasks; BC, breast cancer survivors; OC, other cancer survivors; NC, participants with no history of cancer.

## Data Availability

The KNHANES database is publicly available (https://knhanes.kdca.go.kr (accessed on 29 November 2022) [in Korean]).
